# Disassembly of the fruit cell wall by the ripening-associated polygalacturonase and expansin influences tomato cracking

**DOI:** 10.1038/s41438-018-0105-3

**Published:** 2019-02-01

**Authors:** Fangling Jiang, Alfonso Lopez, Shinjae Jeon, Sergio Tonetto de Freitas, Qinghui Yu, Zhen Wu, John M. Labavitch, Shengke Tian, Ann L. T. Powell, Elizabeth Mitcham

**Affiliations:** 1 Department of Horticulture, Nanjing Agricultural University, Nanjing, 210095 China; 2 Department of Plant Sciences, University of California, Davis, 95616 USA; 3 Gangwon Agricultural Research and Extension Services, Chuncheon, 200-150 South Korea; 4 Institute of Vegetables, Xinjiang Academy of Agricultural Sciences, 830091 Urumchi, China; 5 College of Environmental and Resource Sciences, Zhejiang University, 310058 Hangzhou, China

**Keywords:** Transgenic organisms, Translation

## Abstract

Fruit cracking is an important problem in horticultural crop production. Polygalacturonase (SlPG) and expansin (SlEXP1) proteins cooperatively disassemble the polysaccharide network of tomato fruit cell walls during ripening and thereby, enable softening. A Golden 2-like (GLK2) transcription factor, SlGLK2 regulates unripe fruit chloroplast development and results in elevated soluble solids and carotenoids in ripe fruit. To determine whether SlPG, SlEXP1, or SlGLK2 influence the rate of tomato fruit cracking, the incidence of fruit epidermal cracking was compared between wild-type, Ailsa Craig (WT) and fruit with suppressed *SlPG* and *SlEXP1* expression (*pg/exp*) or expressing a truncated nonfunctional Slglk2 (*glk2*). Treating plants with exogenous ABA increases xylemic flow into fruit. Our results showed that ABA treatment of tomato plants greatly increased cracking of fruit from WT and *glk2* mutant, but not from *pg/exp* genotypes. The *pg/exp* fruit were firmer, had higher total soluble solids, denser cell walls and thicker cuticles than fruit of the other genotypes. Fruit from the ABA treated *pg/exp* fruit had cell walls with less water-soluble and more ionically and covalently-bound pectins than fruit from the other lines, demonstrating that ripening-related disassembly of the fruit cell wall, but not elimination of SlGLK2, influences cracking. Cracking incidence was significantly correlated with cell wall and wax thickness, and the content of cell wall protopectin and cellulose, but not with Ca^2+^ content.

## Crop genetics: Keeping tomato skins together

Researchers in the USA reveal a link between cell wall composition and splitting skin in ripe tomatoes, known as cracking. To unravel the mechanism behind cracking, a team led by Elizabeth Mitcham of the University of California, Davis, compared wild-type tomato plants, mutant plants with increased soluble solids, and mutant plants with altered cell wall structure. They treated the three lines with the hormone ABA to induce cracking and measured several traits in the fruit. ABA treatment did not affect cracking incidence in the cell wall mutant but increased its frequency in the other two lines. Cell wall composition and thickness were the traits with the strongest correlation with cracking rate. Altogether, these findings show that cell wall structure is a major determinant of cracking and point the way towards reducing skin splitting in tomatoes.

## Introduction

Cracking of the epidermis of harvested fruit destroys the appearance and increases the susceptibility of fruit to infections by opportunistic pathogens. Fruit with cracks are not marketable, and, therefore, have reduced economic value. Fissures of the fruit epidermis often occur prior to harvest, but can also occur after harvest, depending on storage and environmental conditions^1,2^. The predisposition to form cracks has been correlated with heredity, various fruit traits (shape, size, firmness, strength and components of pericarp, anatomical structure, water absorbing capacity of the pericarp, number and distribution of stomates, growth period) and external causes, such as cultivation practices (irrigation, nutrition, hormone applications) and growing environment (humidity, temperature, wind, and light)^1,3–10^. Many researchers have attributed cracking predisposition to the thickness of the fruit’s cuticular layer adjacent to the epidermal and sub-epidermal cells^3,11–14^. Cracking has also been linked to the loss of flesh firmness and cell wall integrity^15,16^. Fruit that are susceptible to cracking often have high levels of soluble solids and produce juice with elevated concentrations of osmotically active compounds^17^.

As fruit ripen, there is a dramatic increase in their tendency to crack^13,18^. The production of large, uncracked, ripe fruit in cultivars with thin skins and high soluble solids has proven to be an unmet challenge. The complexity and structural plasticity of the ripening process are challenges for approaches designed to understand the relationship between ripening-associated softening, sugar accumulation and cracking.

Considerable reductions in the incidence and degree of fruit cracking may be achieved by changing cultural or postharvest practices^3,19^. Consistent watering or exogenous applications of boron, calcium and/or growth promoters, such as GA_3_, can reduce cracking. Applications of calcium and boron strengthen the linkages between polysaccharides in the cell wall, increasing firmness^19,20^. Applications of GA_3_ likely decrease cracking because this treatment increases the deposition of cuticular material in the epidermis and makes it more elastic^21,22^. Treating plants with abscisic acid (ABA) increases water movement into and promotes enlargement of the fruit. ABA treatment also increases the tendency of fruit to crack^23^. Application of ABA to “Cabernet Sauvignon” grape berries promotes ripening and the expression of *PG1* and proline-rich cell wall protein genes, typically expressed during ripening^24^.

Cracking in tomato (*Solanum lycopersicum*) fruit most commonly begins as they ripen. During ripening, cell wall modifying proteins, including polygalacturonases (PGs) and expansins (EXPs), cooperatively disassemble wall polysaccharide networks and, thereby, contribute to the softening of fruit. Differences in cell wall structure between varieties and between unripe and ripe fruit could be an important factor in fruit tendency to crack.

Quantitative and qualitative changes in the sugars in ripe fruit could influence water potential and also contribute to the tendency of the fruit to crack. Over-expression of a Golden 2-like (*GLK*2) transcription factor, *SlGLK2*, in tomato enhances chloroplast elaboration and photosynthesis gene expression in developing fruit, and results in ripe fruit with elevated soluble solids content^25,26^.

It is desirable to breed or select for varieties whose fruit resist cracking under diverse environmental conditions without hormone treatments. Therefore, we investigated whether reducing the simultaneous expression of *SlPG* and *SlEXP1* genes affects the tendency of fruit to crack. We were also interested to observe cracking of tomato lines with functional or non-functional forms of *SlGLK2* to explore the contributions of solutes and sugars to the fruit's predisposition to form cracks. ABA was used as a tool to enhance cracking incidence of the tomato fruit.

## Materials and methods

### Plant material

A preliminary experiment was conducted in 2012, followed by a similar but more extensive experiment in 2013 with 3 genotypes. The Alisa Craig *S. lycopersicum* cultivar (hereafter WT) (LA3736) expresses functional *SlPG*, *SlEXP1* and *SlGLK2* genes. The transgenic line, *pg/exp*, has suppressed ripe fruit expression of both *SlPG* and *SlEXP1*. It was obtained by crossing homozygous AC *SlPG-*suppressed and *SlEXP1-*suppressed lines. Suppression of *SlPG* or *SlEXP1* alone did not significantly enhance fruit firmness. However, fruits with suppressed expression of both genes were significantly firmer throughout ripening with a long-term storage and more viscous juice than control fruits^27^. The monogenic *u/u* mutant of AC, “Craigella” (LA3247, hereafter *glk2*), contains a mutation in *SlGLK2* that results in a truncated and, therefore, nonfunctional *glk2* (*u*) protein^25^.

In the 2012 experiment, plants of the *pg/exp* and *glk2* genotypes were grown from 15 December 2011 to 3 May 2012 in greenhouses at the University of California, Davis. Prior to germination, seeds were soaked for 3 h in water and for 30 min in a 10% solution of bleach to reduce potential viral contamination, then washed 3 times with deionized water and placed into Petri dishes with 7 mL 30 µM GA_3_ for 2 days at 4 °C. Subsequently, seeds were germinated in a growth chamber at 25 °C. Seedlings were transplanted and moved to the greenhouse on 16 January. There were 64 plants of each genotype (*pg/exp* or *glk2*) subdivided into two treatments (water or ABA) and 4 replications with 8 single plant replicates per treatment. Seedlings were grown in 9.5-L pots containing 33.3% each peat, sand, and red wood compost with 2.6 kg dolomite lime m^−3^. The plants were irrigated twice per day with 350 mL of UC Davis nutrient solution containing NH_4_^+^(6 ppm), NO_3_^−^ (96 ppm), H_2_PO_4_^−^ (26 ppm), K^+^ (124 ppm), Ca^2+^ (90 ppm), Mg^2+^ (24 ppm), SO_4_^2−^ (16 ppm), Fe (1.6 ppm), Mn (0.27 ppm), B (0.25 ppm), Cu (0.16 ppm), Zn (0.12 ppm) and Mo (0.016 ppm). Plants were pollinated on 8 March 2012, and were topped on 15 March 2012 when they had 2 clusters of flowers. On 18 April, ABA and control spray treatments began. The plants were sprayed 1× per week for 3 weeks with a backpack applicator until the plants were completely covered with a solution containing deionized water (control) or 0.5 mg L^−1^ ABA (Valent Biosciences, Clovis, CA); each solution also contained 0.5 mL L^−1^ polysorbate 20 (Tween^®^20, Fisher Scientific, Fair Lawn, NJ) as a surfactant. The cracking fruits were counted and cracking rates were calculated on 26 April. The other characteristics of the fruits were then analyzed.

In 2013, WT, *pg/exp* and *glk2* plants were grown from 17 December 2012 to 6 May 2013 in greenhouses. Seed germination protocols were like those used in 2012. Seedlings were transplanted into pots in the greenhouse on 14 January. There were 192 plants in total with 64 plants for each genotype, as in 2012. In the greenhouse, passive ventilation was used to maintain a relative humidity of 26.1–27.4%. The average temperature ranged from 21.5 to 22.7 °C with minimum of 12.8 °C and maximum of 35.0 °C. Cultivation practices were the same as in 2012, although the irrigation schedule was modified due to growth periods. Plants were initially irrigated twice per day with 350 mL of UC Davis nutrient solution. The irrigation frequency was increased to 5 times per day with 200 mL at full bloom (4 March). It was then increased to 8 times per day the week after pollination (18 March). Irrigation started 1 h before sunrise and finished 1 h after sunset. Three days before harvest, the irrigation frequency was adjusted to one time per day with 4800 mL water (starting at 11:00 am) to further enhance cracking. Plants were topped on 28 February when they had 2 clusters of flowers and were pollinated on 1, 4, 8, and 11 March. On 18 March, the spray treatments with water or ABA began, applied 3 times per week for 7 weeks, until 22 April. Tomato cracking rate, firmness, total soluble solids (TSS) and titratable acidity (TA) were analyzed on 30 April. The fruit materials for other analyses were preserved until the next day and then analyzed.

### Tomato fruit size and expansion rate during development

In 2012, fruit diameters were measured using a caliper on 26 April. In 2013, fruit that were approximately equal in size at the start of treatment application (diameters 18.4 minimum to 19.1 mm maximum) were selected for analysis and tagged. The diameters of 12 fruits per treatment and genotype were measured every 2–3 days, beginning when the first treatment was applied and continuing until the fruit were ripe. The expansion rate was calculated by dividing the increase in diameter in 2 or 3 days by the number of growth days.

$${{\mathrm{Expansion}}\,{\mathrm{rate = }}\frac{{{\mathrm{The}}\,{\mathrm{increase}}\,{\mathrm{of}}\,{\mathrm{diameter}}\,{\mathrm{in}}\,{\mathrm{2}}\,{\mathrm{or}}\,{\mathrm{3}}\,{\mathrm{days}}}}{{{\mathrm{growth}}\,{\mathrm{day}}}}}$$

### Stomatal conductance

Stomatal conductance was measured on 19 April (1 d after the first spray treatment application) in 2012 and on 21 and 28 March in 2013 (3 and 10 days after the first treatment) in 2 fully expanded leaves located on opposite sides of each plant (fifth to seventh basipetally located leaves). Measurements were made between 1:00 and 3:00 p.m. with a steady-state porometer (LI-1600, LI-COR Biotechnology, Lincoln, NE).

### Percent cracking

In 2012, the incidence of cracking was determined visually on 26 April in all full-sized fruit which were then categorized into different ripeness stages from the same or different clusters based on external fruit color and tagged date. In 2013, the incidence of cracking of fruit at the MG, turning and RR stages were recorded on 30 April.

### Measurement of fruit firmness, total soluble solids, and titratable acidity

In 2012, fruit firmness was measured on turning, pink and RR fruit, which had no cracks on 2 May. In 2013, fruit firmness was measured on fruit with no cracks at the MG and RR stages on 30 April. In both years, firmness was measured by compressing the fruit at the equator using a 51 mm flat stainless steel probe (test speed 2 mm s^−1^) (TaXT2i Texture Analyzer; Texture Technologies). In 2012, fruit were compressed 5 mm and in 2013 fruit were compressed 2 mm.

TSS and TA were measured on RR fruit on 26 April in 2012. In 2013, TSS and TA were measured in cracked RR fruit and uncracked fruit at the MG and RR stages on 30 April. For each genotype, treatment, and phenotype, four fruit were cut in half from peduncle to blossom-end, creating 8 replicated samples. The samples were squeezed and the juice was filtered through 2 layers of cheesecloth. A few drops of juice were used to measure TSS content by refractometry (Reichert AR6 series). Four grams of juice diluted in 20 mL deionized water were titrated (Radiometer Titralab Tim 850 titration manager and SAC80 sample charger) to determine TA based on citric acid equivalents.

### Isolation of cell walls

The preparation of total cell walls (i.e., alcohol-insoluble residue, AIR) followed the protocols of Vicente^28^; the AIR was dried and was identified as the total cell wall fraction of ground tissue. Samples of approximately 15 g of exocarp (collected from peduncle to blossom-end portions on the fruit) and mesocarp (collected from peduncle to blossom-end portions), peduncle (peduncle proximal half, including exocarp and mesocarp) or blossom-end (blossom-end half, including exocarp and mesocarp) tissues from fruit harvested RR with no cracks were used for cell wall extractions.

### Pectin and cellulose contents

Fractions enriched for pectic polymers of the isolated cell wall preparations were sequentially extracted from AIR. From 200 mg of AIR, water soluble pectins (WSP, fraction mainly pectins with no strong bonds to the rest of the cell wall), chelator soluble pectins (CSP, fraction of pectins that were ionically bound into the wall via linkages to Ca^2+^ and are soluble in the chelator *trans*-1, 2-diaminocyclohexane-*N*,*N,N*',*N'*-tetraacetic acid (CDTA)) and sodium carbonate-soluble pectins (SSP, pectin extracted using 50 mM Na_2_CO_3_, a fraction containing mainly pectins covalently bound by ester linkages into the cell wall) were prepared, as described in Vicente^28^. Cellulose was measured following the protocols of Vicente too^28^.

### Calcium content

The dried AIR samples of the tomato fruit analyzed in 2013 were weighed and ground to a uniform powder. The calcium content of 200 mg of the replicated AIR preparations was determined by inductively coupled plasma optical emission spectrometry (Optima 2100 DV, Perkin Elmer, America)^29^.

### Preparation of plant materials for microscopy

Samples of RR fruit were fixed for microscopic evaluation. Sections with exocarp and mesocarp (1 mm × 1 mm × 2 mm) were cut from the peduncle half of the fruit using razor blades and the tissues were immediately fixed in PEM buffer (50 mM PIPES, 5 mM EGTA, and 5 mM MgSO_4_, pH 6.9) containing 4% (w/v) paraformaldehyde under vacuum (1 h at room temperature). Tissues were dehydrated through a graded ethanol series, and then infiltrated with LR White resin (Sigma-Aldrich, St. Louis, MO, USA) and 95% ethanol (1:1, v/v) for 40 min, followed by 100% resin for 40 min, and then the samples were held in 100% resin overnight on a rotator at room temperature. The samples were polymerized in gelatin capsules for 2 days at 0 to −20 °C in a cryo chamber. Sections were cut to a thickness of 1 µm using a glass knife on an Ultra cut microtome (Leica), and then collected on multiwell slides (ICN Biomedicals) filled with water.

### Immunofluorescence microscopy

For reactions with the JIM5 and JIM7 antibodies^30,31^, fruit tissue sections that had been fixed and embedded were incubated in 1× phosphate buffered saline (PBS, pH7.2, Sigma-Aldrich) with 0.05% Tween-20 containing 5% (w/v) nonfat milk protein (MP/PBS, blocking solution) for 1 h. The blocking solution was removed and one drop (approximately 30 µL) of primary antibody (e.g., JIM5) diluted 1:10 in MP/PBS was added to the tissue and incubated overnight. Afterwards, samples were washed in MP/PBS (10 min) twice and incubated with a 100-fold dilution of FITC-conjugated rabbit anti-rat IgG (whole molecule; batch no. 078K4833, Sigma-Aldrich) in MP/PBS for 1 h in darkness. After washing in PBS twice for 5 min each to remove the excess second antibody, the sections were mounted in an anti-photo-bleaching medium (100 mM Tris, pH 9.2, 50% glycerol and 1 mg mL^−1^ *p*-phenylenediamine), before sections were examined under blue light (488 nm) using an Olympus BH2 microscope. The samples of each genotype were divided into four groups: one for incubation with a monoclonal cell wall antibody (primary antibody JIM5 or JIM7) and the corresponding FITC-conjugated secondary antibody; the other three (with only primary, secondary or no antibody) for three types of experimental controls. Images were all obtained following exposure for exactly 3 s to guarantee that any differences observed were caused by treatment or genotypes, rather than by differing exposure times. The relative fluorescent intensity from three slices was measured from three visible regions in each slice for each treatment using the ImageJ software.

### Transmission electron microscopy

Samples were prepared according to Zhang^32^. Ultrathin fixed and embedded fruit tissue sections were examined with a Hitachi-7650 transmission electron microscope. The cell wall thickness from 5 slices was measured from six visible regions in each slice for each treatment.

### Light microscopic observation

For observation of the cuticular wax layer, toluidine blue O (TBO) staining, fresh fruit sections were incubated in a solution of 1% (w/v) TBO containing 1% (w/v) sodium borate for 5 min, washed in water (3 min), mounted in glycerol, and observed using an Olympus BH2 microscope. TBO stains waxes preferentially. The thickness of the cuticular wax was determined from 5 slices and 6 views in each slice for each treatment.

### Data analysis

Analysis of variance (ANOVA) to determine differences in fruit characteristics between the genetic materials and treatments, and correlations between fruit parameters and cracking incidence was determined using statistical product and service solutions (SPSS) 16.0 (SPSS Inc. Chicago, IL, USA).

## Results

### Tomato fruit size and expansion rate during development

**Fig. 1:**
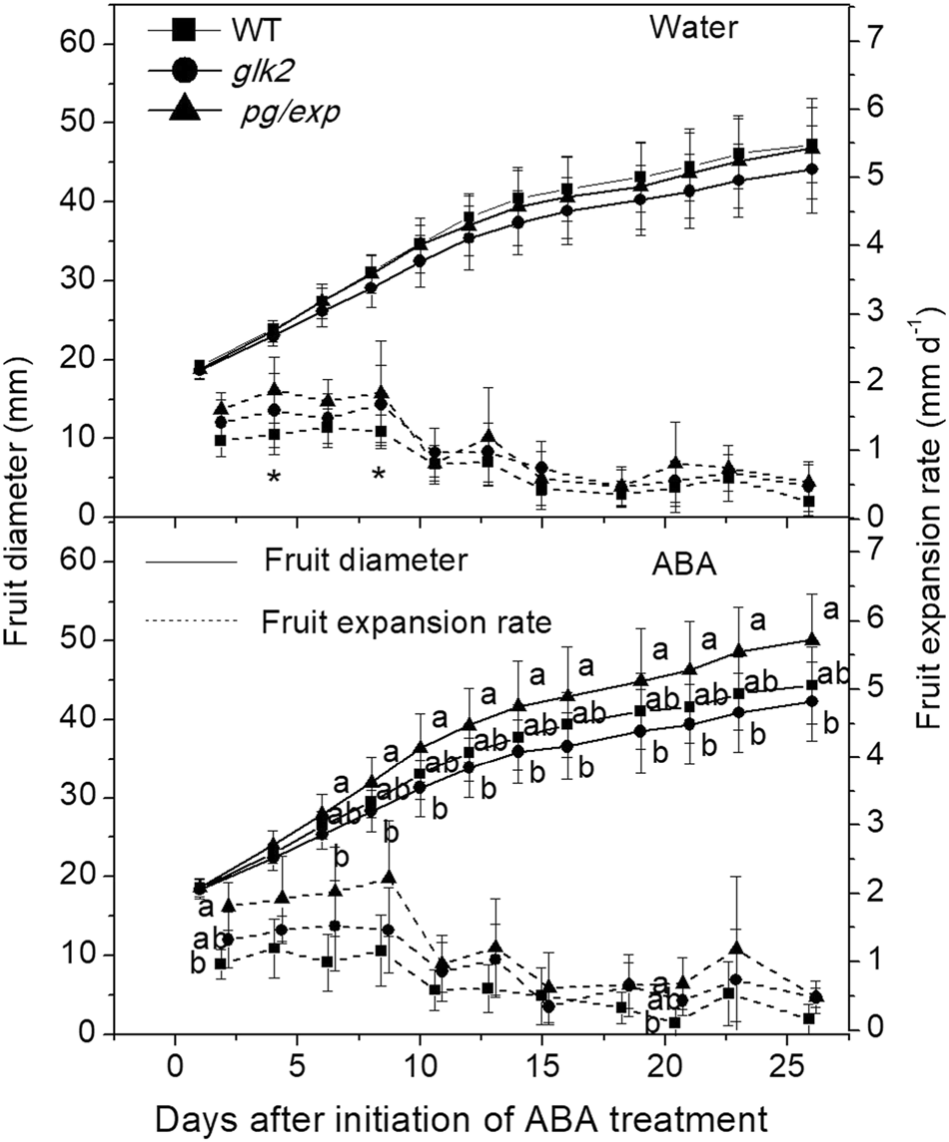
Increase in diameter (solid line) and expansion rate (dashed line) of WT, *glk2*, and *pg/exp* ripe tomato fruit following initiation of water and ABA treatment at 18 mm average fruit size. WT indicates wild type Alisa Craig; *glk2* indicates a nonfunctional *Slglk2* mutant; and *pg/exp* indicates transgenic fruit with suppressed *SlPG* and *SlEXP1* expression, the same as below. Different lowercase letters indicate a significant difference between genotypes (<0.05). Differences between the water and ABA treatment within the same genotype were determined with a *T* test. Asterisk (*) indicates a significant difference between water and ABA treatments (<0.05)

The size and the rates of expansion of the diameters of the fruit while attached to the plant were measured during development to determine whether the genotypes or the ABA treatments altered the fruit characteristics. In 2012, there was no difference in the size of the *pg/exp* fruit (52.7 mm in ABA treated plants and 52.2 mm in water treated plants) compared to the *glk2* mutant fruit (54.1 mm in ABA treated plants and 52.8 mm in water treated plants). In 2013, the initial fruit diameters (day 1) were similar (18.6 mm, 18.6 mm and 18.4 mm, for the WT, *glk2* mutant and *pg/exp* genotypes, respectively). Fruit on plants treated with water (control), showed no significant differences between genotypes (Fig. 1). After spraying with ABA for 26 days, the *pg/exp* fruit averaged 50.1 mm in diameter; and the diameters of the WT (44.4 mm) and *glk2* mutant (42.3 mm) fruit were clearly smaller (*P* < 0.05), and the rate of expansion of *pg/exp* (around day 3 and 20) was the highest among the 3 genotypes.

### Stomatal conductance

**Fig. 2:**
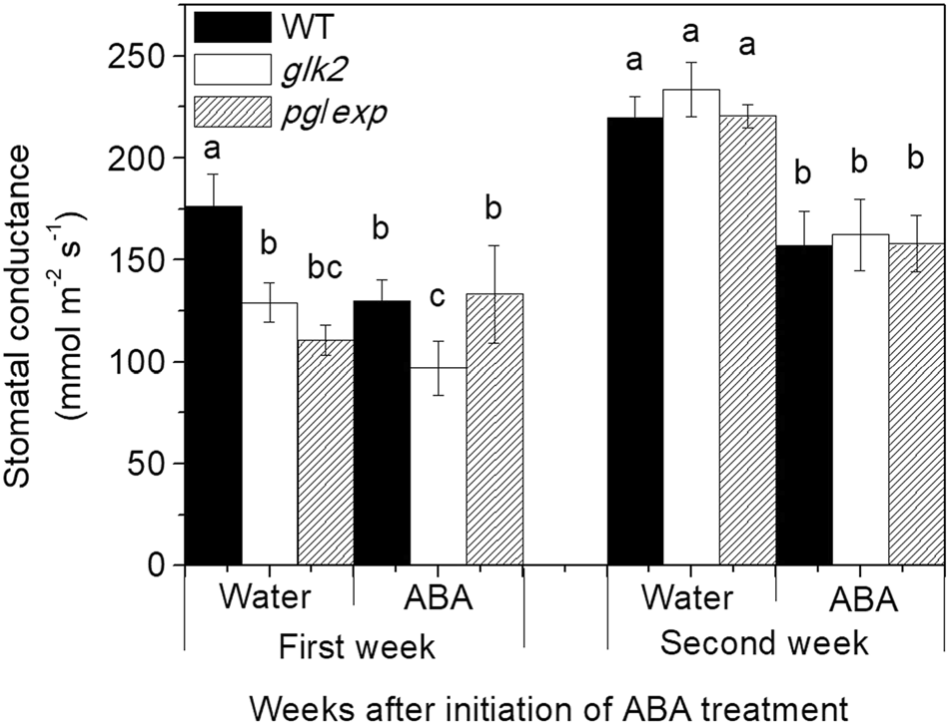
Stomatal conductance of WT, *glk2*, and *pg/exp* tomato fruit following initiation of water and ABA treatment at 18 mm average fruit size. Different lowercase letters indicate a significant difference in stomatal conductance in the same week (<0.05)

In 2012, stomatal conductance for *pg*/*exp* and *glk2* mutant was 171.4 and 183.3 mmol m^−2^ s^−1^, respectively, in water treated plants, and conductance decreased to 53.9 and 31.4 mmol m^−2^ s^−1^ in ABA treated plants. In 2013, results were similar. Spraying plants of all genotypes with ABA decreased stomatal conductance for up to two weeks of treatments (*P* < 0.05) (Fig. 2), but no further decrease was observed compared to water-sprayed plants (data not shown). There were no consistent differences in stomatal conductance between genotypes, although in the first week of spraying, leaves from WT plants had slightly higher stomatal conductance than *glk2* mutant leaves (*P* < 0.05).

### Percent cracking

**Fig. 3:**
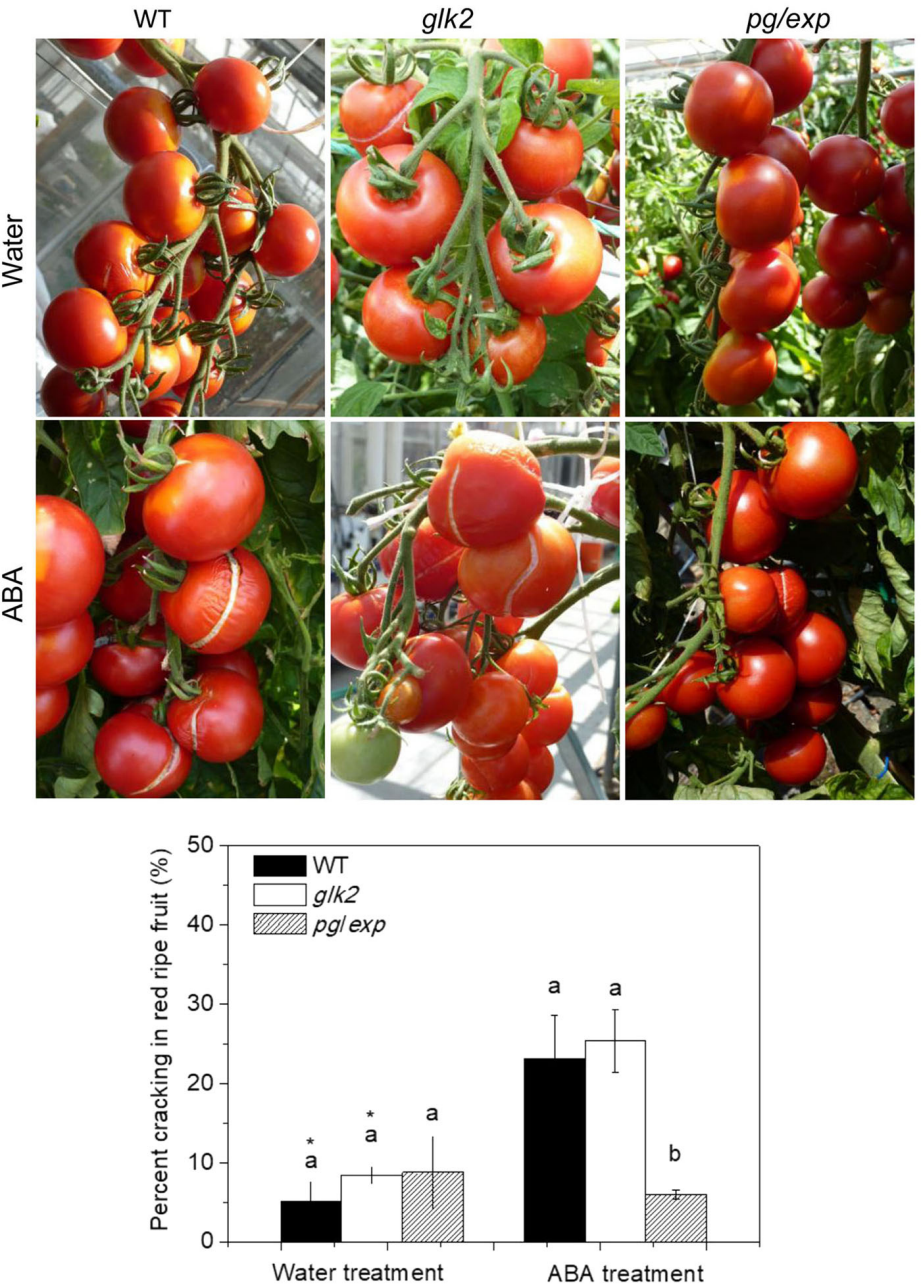
Percent cracking in red ripe WT, *glk2*, and *pg/exp* tomato fruit treated with water or ABA beginning at 18 mm average fruit size. Different lowercase letters indicate a significant difference between genotypes (<0.05). Difference between the water and ABA treatment within the same genotype were determined with a *T* test. Asterisk (*) indicates a significant difference between water and ABA treatments (<0.05). The same as below

The incidence of cracking, as evidenced by visible fissures in the epidermis, increased as fruit ripened from MG to turning to RR stages in 2012 and 2013. In both years, no MG fruit were cracked and less than 5% of turning fruit were cracked, regardless of the treatment (data not shown). In 2012, ABA treatment increased the incidence of cracking of the RR *glk2* mutant tomatoes (30.2% of ABA treated plants compared to 20% of water treated plants), but not the *pg/exp* tomatoes (13.1% in ABA treated plants and 12.1% in water treated plants). Similarly, in 2013, treatment of the plant with ABA increased the incidence of cracking of RR fruit of the WT and *glk2* mutant genotypes, but not of the *pg/exp* genotype (Fig. 3).

### Fruit firmness, total soluble solids, and titratable acidity

To test the hypothesis that fruit texture and the resistance to tension indicate the tendency of fruit to crack, fruit firmness was measured. In 2012, firmness decreased as the fruit ripened and the *pg/exp* fruit were firmer than *glk2* mutant. The firmness of the non-cracked, ABA treated *pg/exp* tomatoes were 1.31, 1.31, and 1.09 times greater than that of the *glk2* mutant tomatoes on turning, pink and RR stages, respectively. In 2013, the firmness of MG and RR fruit without cracks was measured on the day of harvest. As in 2012, firmness of MG fruits was greater than RR fruits for each genotype (Table 1) (*P* < 0.05). And the firmness of ABA treated *pg/exp* tomatoes were 1.19 and 1.10 times greater than that of the *glk2* mutant, and 1.13 and 1.20 times greater than that of the WT fruit on MG and RR stages, respectively (Table 1).

**Table 1:** Total soluble solids, titratable acidity and firmness of mature green, red ripe uncracked and red ripe cracked tomatoes of three genotypes (2013)

Stage	Genotypes	Total solublesolids (%)		Titratable acidity (g L^−1^)		Firmness (*N*)	
		Water	ABA	Water	ABA	Water	ABA
Mature green	WT	4.82 ab	4.53 ab	0.93 a	0.87 a	6.13 b	6.50 b
	*glk2*	4.49 b	4.35 b	0.79 b	0.70 b	6.27 b	6.20 ab
	*pg/exp*	5.15 a^a^	4.61 a	0.88 ab	0.83 ab	7.37 a	7.37 a
Red ripe	WT	5.68 b^a^	4.89 b	0.51 a^a^	0.40 a	1.20 b	1.58 b
	*glk2*	5.16 b^a^	4.80 b	0.41 a	0.37 a	1.72 ab	1.73 ab
	*pg/exp*	6.46 a^a^	5.58 a	0.50 a^a^	0.40 a	2.03 a	1.90 a
Red cracked	WT	5.45 b	4.90 a	0.50 a^a^	0.44 a	–	–
	*glk2*	4.97 b	5.01 a	0.45 a	0.47 a	–	–
	*pg/exp*	6.35 a^a^	5.58 a	0.45 a	0.42 a	–	–

Different lowercase letters indicate a significant difference between genotypes within a stage and treatment (water or ABA) (*P* < 0.05)

^a^Indicates a significant difference between water and ABA treatments (*P* < 0.05)

In 2012, the *pg/exp* fruit had consistently higher TSS (5.90% in ABA treated and 5.78% in water treated plants) than the *glk2* mutant (5.48% in ABA and 5.10% in water treated plants). Similar results were observed in 2013 (Table 1). The *pg*/*exp* fruit had consistently higher TSS than the *glk2* mutant and WT fruit except for ABA-treated red ripe cracked fruit. The TSS increased in fruit of all genotypes as they ripened from MG to RR. Fruit of the same genotype and the same ripeness stage from plants treated with ABA had lower TSS and TA than fruit from plants treated with water. At the MG stage, WT fruit had higher TA than the *glk2* mutant fruit (*P* < 0.05).

### Pectin and cellulose contents

**Fig. 4:**
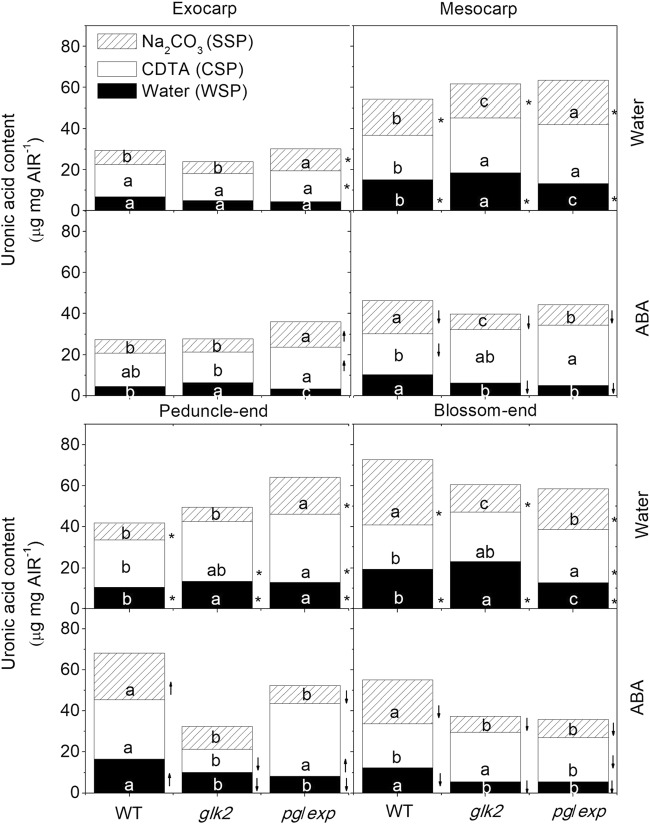
Water, CDTA, and Na_2_CO_3_-solubilized pectins (WSP, CSP, SSP) as measured by the uronic acid equivalents in alcohol-insoluble residues (AIR) prepared from the exocarp and mesocarp portion of red ripe WT, *glk2* and *pg/exp* tomato plants treated with water or ABA. ↑ indicated that pectin (WSP, CSP, or SSP) from ABA-treated portion increased compared to water treated portion, ↓ indicated that pectin (WSP, CSP or SSP) from ABA-treated portion decreased compared to water treated portion

To determine whether differences in the composition of the fruit cell wall polysaccharide fractions could account for differences in fruit cracking among the genotypes, AIRs were prepared from the exocarp, mesocarp, peduncle, and blossom-end portions of the fruit at the RR stage. In this analysis, pectin solubilization is indicated primarily by the proportion of galacturonic acid containing polysaccharides in each fraction. The WSP fraction was reduced in *pg/exp* compared to WT and *glk2* mutant fruit in nearly all parts of the fruit except the exocarp and peduncle portions of water treated plants (Fig. 4). The CSPs were greater in the mesocarp portion of the ripened *pg/exp* fruit than the WT fruit in both water and ABA treatments, and was greater in the peducle-end and blossom-end tissues of *pg*/*exp* fruit compared to WT after water treatment. The SSP fractions were also more abundant in *pg/exp* than in WT and *glk2* mutant fruit in the exocarp of both water and ABA treated fruits as well as in the mesocarp and peducle-end water treated fruits. But in some cases, the WT fruit had a higher proportion of SSP (Fig. 4). And ABA-treated fruits had less WSP, SSP, and total pectin than did water treated fruits in the mesocarp and blossom-end portion of fruits (*P* < 0.05) (Fig. 4).

**Fig. 5:**
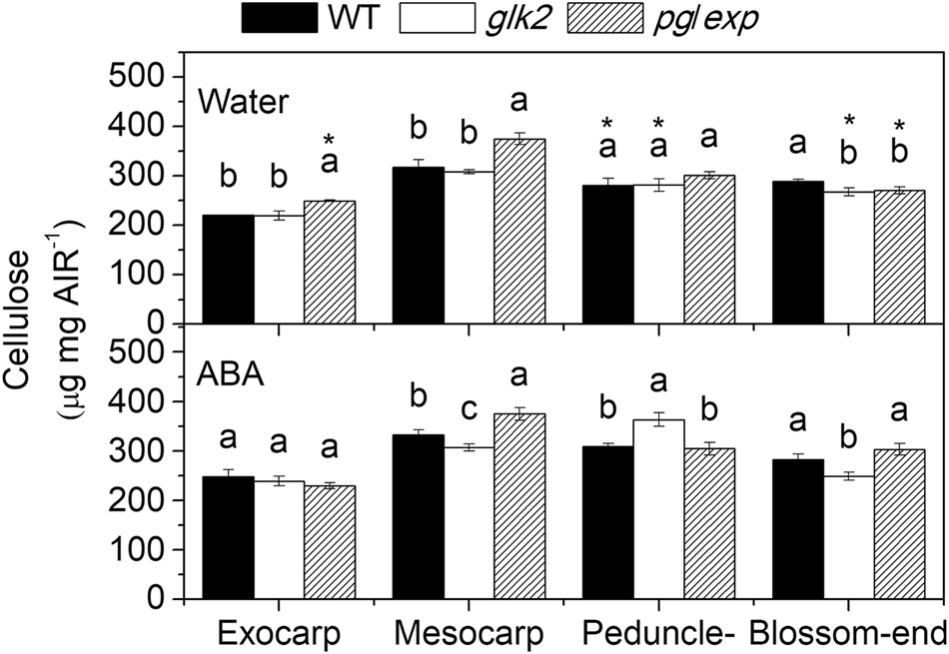
Cellulose content in the alcohol-insoluble residues (AIR) prepared from the exocarp and mesocarp portion of red ripe WT, *glk2*, and *pg/exp* tomato fruit from plants treated with water or ABA

The cellulose content of the extracted cell walls was higher in the mesocarp portion of *pg/exp* fruit than in WT and *glk2* mutant fruit (*P* < 0.05) (Fig. 5).

### Calcium content in fruit and the cell walls of fruit

The Ca^2+^ content of *pg/exp* fruit and their cell walls was lower than that of WT fruit, whether the plants were treated with water or with ABA, and also lower than that of *glk2* mutant fruit from water treated plants. The Ca^2+^ content of WT and *pg/exp* fruit treated with ABA was higher compared to fruit from plants treated with water, while the Ca^2+^ content in *glk2* mutant fruit from plants treated with ABA was lower than that of fruit from water-treated plants (*P* < 0.05) (Table 2).

**Table 2:** Ca^2+^ content (µg g^−1^) in fruits and cell wall of fruits of three genotypes treated with water or abscisic acid (ABA)

Genotypes	Fruits		Cell wall	
	Water	ABA	Water	ABA
WT	10.22 ± 0.62b^a^	14.43 ± 0.49a	39.27 ± 1.46b^a^	53.91 ± 1.50a
*glk2*	15.68 ± 0.11a^a^	10.97 ± 0.15b	50.03;± 1.44a	40.87 ± 5.47b
*pg/exp*	9.17 ± 0.13c^a^	9.92 ± 0.33c	33.37 ± 0.86c^a^	37.60 ± 1.61b

Differences between the genotypes were determined with a Tukey test. Different lowercase letters indicate a significant difference between genotypes within water or ABA treatment (<0.05). Difference between the water and ABA treatment within the same genotype were tested with a *T* test

^a^Indicates a significant difference between water and ABA treatments (<0.05)

### Pectin esterification and cracking

**Fig. 6:**
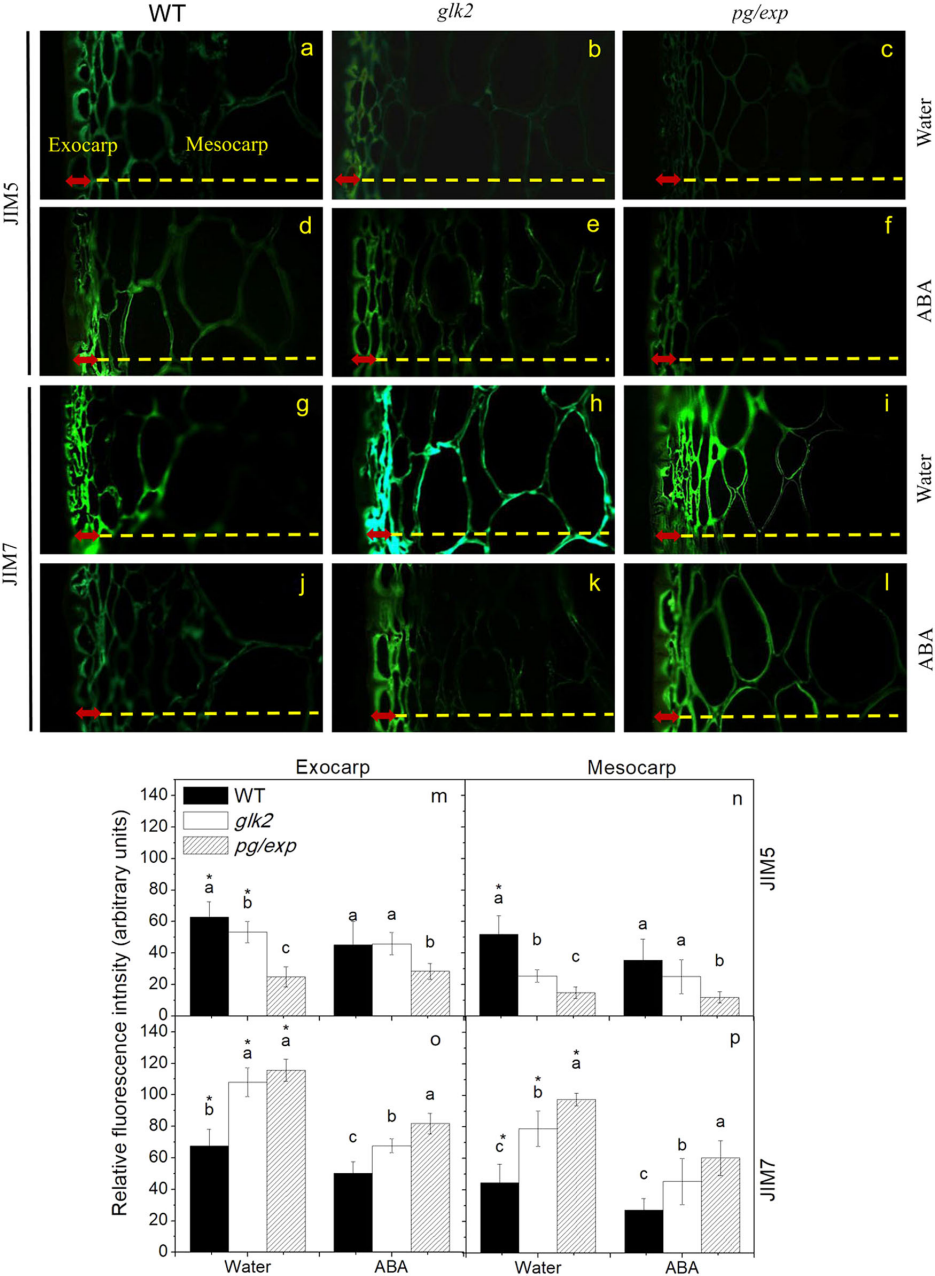
Indirect immunofluorescence detection of pectin esterification in red ripe WT (**a**, **d**, **g**, **j**), *glk2* (**b**, **e**, **h**, **k**), and *pg/exp* (**c**, **f**, **i**, **l**) tomato fruit from plants treated with water (**a**–**c**, **g**–**i**) or ABA (**d**–**f**, **j**–**l**). Sections from fruits of each genotype and treatment were analyzed with two monoclonal antibodies, JIM5 (identifies HG pectins with low levels of methyl esterification) (**a**–**f**, **m**, **n**) and JIM7 (identifies HG pectins with high levels of methyl esterification) (**g**–**l**, **o**, **p**). Red arrows indicate exocarp and yellow dotted line represents mesocarp

Indirect immunofluorescence microscopy was used to detect two classes of pectins in microscopic sections of RR tomato fruit. The monoclonal antibody, JIM5, identifies pectin with low levels of esterification and JIM7, detects highly esterified pectin^30,33^. The JIM5 antibody weakly recognized epitopes in WT and *glk2* mutant fruit cell walls, but there was even less JIM5 recognition of homogalacturonan components in *pg/exp* fruit, regardless of treatment (Fig. 6a–f, m, n). These differences in antibody binding were particularly apparent in the mesocarp portion of the fruit. The JIM7 antibody strongly recognized esterified pectins in fruit from all genotypes from water treated plants (Fig. 6g–i, o, p), but fewer epitopes were seen in fruit from ABA-treated plants (Fig. 6j–l, o, p). However, JIM7 recognized esterified pectins most strongly in *pg/exp* and least strongly in WT fruit (Fig. 6g, i, j, l–p).

### Cell wall thickness and epidermal waxes

**Fig. 7:**
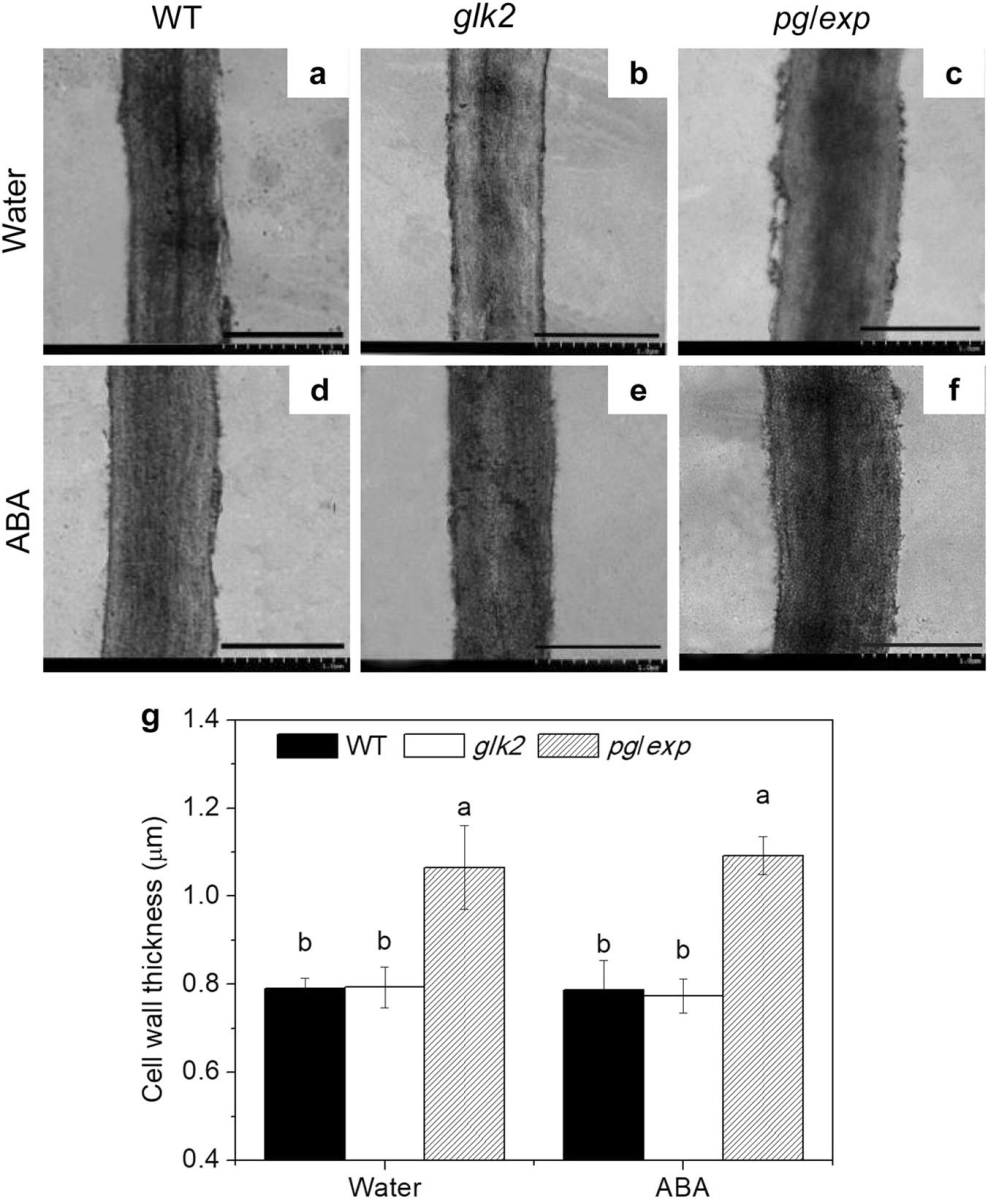
Cell wall density (upper panels) and thickness (lower panels) observed by transmission electron microscopy examination of red ripe WT, *glk2* and *pg/exp* tomato fruit from plants treated with water or ABA. Bar in each section equals 1.0 µm

**Fig. 8:**
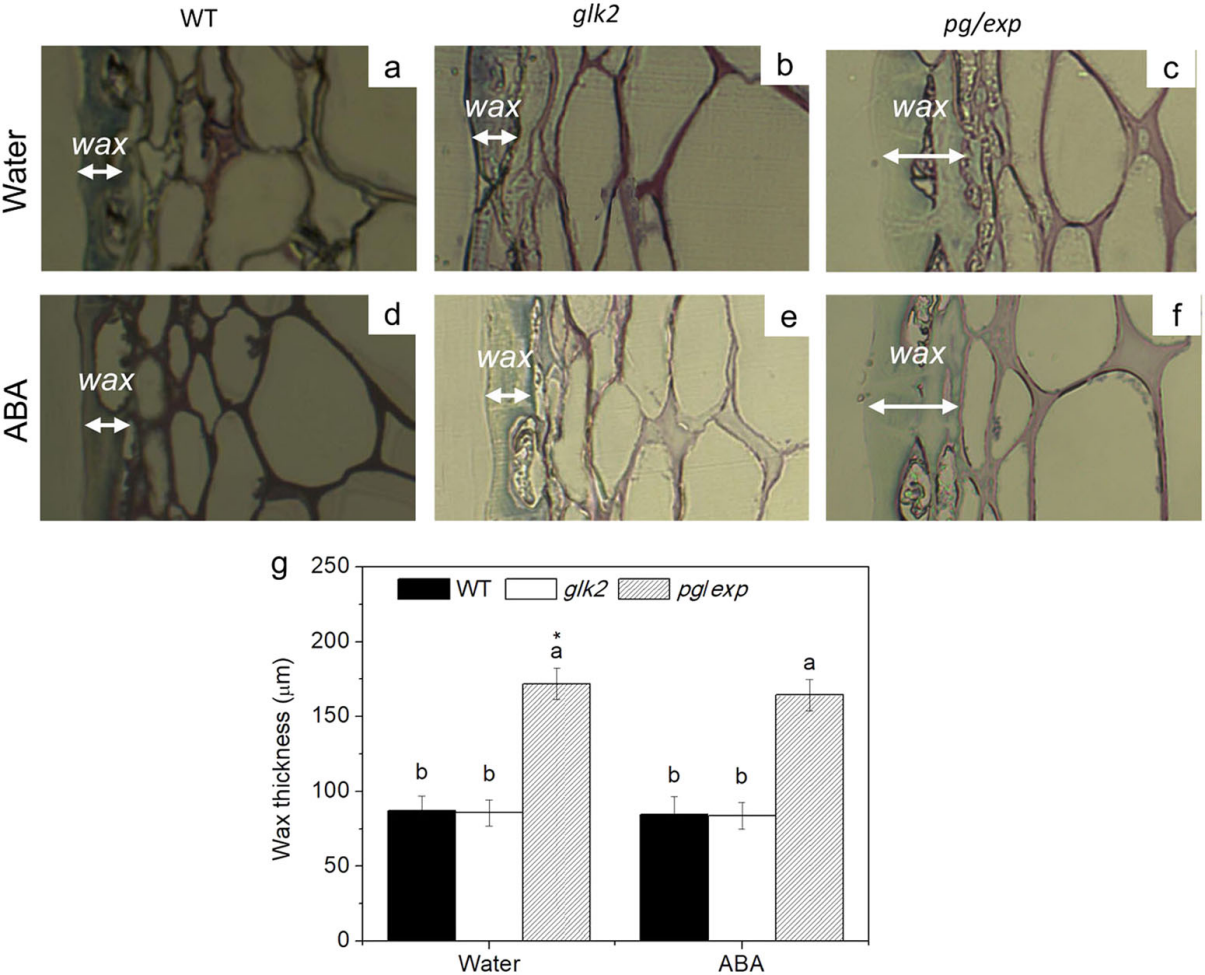
Microscopic inspection of the cuticles of red ripe tomato fruit from WT, *glk2*, and *pg/exp* fruit from plants treated with water or ABA. Microscopy was used to detect epidermal waxes which were stained with toluidine blue O. The white arrows indicate the thickness of the wax

When examined by electron microscopy, cell walls from RR *pg/exp* fruit appeared thicker and denser (Fig. 7c, f) than cell walls of RR WT (Fig. 7a, d) and *glk2* (Fig. 7b, e) fruit. The *pg/exp* fruit also had a thicker wax layer than the other tested genotypes from plants treated with water (Fig. 8a–c) and ABA (Fig. 8d–f).

### Correlation analysis between crack incidence and fruit characteristics

The correlation analysis illustrated that cracking incidence was significantly associated with stomatal conductance (SC1 −0.589*; SC2 −0.582*), cell wall thickness (CW-thickness −0.482*), wax-thickness (−0.497*), and the content of TSS (MG −0.509*; RR −0.614**; RC –0.472*), TA (RR −0.516*), protopectin (Me-SSP −0.477*; Pe-CSP −0.573*; Ex-JIM7P −0.547**; Me-JIM7P −0.569**), and cellulose (Ex 0.491*; Pe 0.724**; Bl −0.495*) (Table 3). However, cracking incidence was not associated with fruit-Ca^2+^ or CW-Ca^2+^.

**Table 3:** The correlation coefficients between crack rate and relevant index

	MG-TSS	RR-TSS	RC-TSS	MG-TA	RR-TA	RC-TA	SC1	SC2	CW-thickness	Wax-thickness		
Crack rate	**−0.509** ^a^	**−0.614** ^b^	**−0.472** ^a^	**−0.391**	**−0.516** ^b^	0.135	**−0.589** ^a^	**−0.582** ^a^	**−0.482** ^a^	**−0.497** ^a^		
	MG-firmness	RR-firmness	Fruit-Ca^2+^	CW-Ca^2+^	Ex-cellulose	Me-cellulose	Pe-cellulose	Bl-cellulose	Ex-JIM5P	Me-JIM5P	Ex-JIM7P	Me-JIM7P
Crack rate	−0.283	−0.047	0.452	0.293	**0.491** ^a^	-0.367	**0.724** ^b^	**−0.495** ^a^	0.121	0.291	**−0.547** ^b^	**−0.569** ^b^
	Ex-WSP	Ex-CSP	Ex-SSP	Ex-SUMP	Me-WSP	Me-CSP	Me-SSP	Me-SUMP				
Crack rate	0.166	−0.239	−0.393	−0.348	−0.43	−0.263	**−0.477** ^b^	**−0.583** ^a^				
	Pe-WSP	Pe-CSP	Pe-SSP	Pe-SUMP	Bl-WSP	Bl-CSP	Bl-SSP	Bl-SUMP				
Crack rate	0.334	**−0.573** ^a^	0.432	−0.099	−0.458	0.026	−0.325	−0.433				

MG-, RR- represent different maturation periods. *MG* mature green, *RR* red ripe; *RC* red ripe cracked fruit, Ex-, Me-, Pe-, Bl- represent different parts of the fruit. *Ex* exocarp, *Me* mesocarp, *Pe* peduncle, *Bl* blossom-end, *CW* cell wall, *TSS* total soluble solids, *TA* titratable acidity, *SC1* stomatal conductance during the first week, *SC2* stomatal conductance during the second week; WSP, CSP, SSP and SUMP indicate water, CDTA, and Na_2_CO_3_-solubilized pectin and the sum of pectin, respectively; JIM5P = Homogalacturonan (HG) pectins with low levels of methyl esterification; JIM7P represents HG pectins with high levels of methyl esterification

^a^Indicates significant correlation (*P* values < 0.05)

^b^Indicates highly significant correlation (*P* values < 0.01)

## Discussion

Previous research indicates that heritable resistance to cracking can be identified in some tomato breeding lines. However, no single genetic locus seems to be responsible for inheritance of the fruit cracking trait and many genes may contribute to the phenotype^2^. Many studies point to the involvement of cell wall structure and possibly the cuticle layer in fruit cracking^1,34–40^. As cell wall networks weaken with fruit ripening^41,42^, even as cell turgor falls, resistance to stresses at the fruit surface may require a greater contribution from wax and cuticle layer structures than they can provide; and cell turgor pushing the plasmalemma against the cell wall also creates some stress on the cell wall polysaccharide networks that may be accommodated by the elasticity of the wall "fabric"; this then leading to cracking.

Polysaccharides make up more than 90% of the mass of the plant cell wall. The pectins are relatively uronic acid-rich polymers that are the most structurally complex polysaccharides in plant primary cell walls^43,44^. PG is believed to be responsible for a large part of the HG pectin depolymerization in ripening tomatoes; PG mRNA, protein, and activity accumulate to very high levels late in the ripening of tomato fruit^34^. Brummell reported that suppression of the ripening-related EXP-encoding gene slowed tomato fruit softening early in ripening, and they hypothesized that EXP1-mediated relaxation of the wall structure is necessary to allow PG or other enzymes access to polyuronide or other wall substrates^38^.

To investigate how PG and EXP may work collaboratively to affect the susceptibility of tomato fruit to cracking, we investigated differences in cell wall composition as influenced by the *pg/exp* genotype in fruit stressed by increased water uptake following treatment with ABA.

Ripening in tomato is accompanied by a shift in pectins from the CSP and SSP to the WSP^45^. The clearest impact of simultaneous suppression of PG and EXP was that the *pg/exp* fruit displayed a substantially reduced breakdown of the cell wall pectin network as they proceed through ripening and the fruit soften less than WT fruit^46^. The pectin polymers in ripe fruit of the *pg/exp* genotype are bigger than those in ripe WT fruit, and there was more SSP in *pg/exp* fruit compared with WT^27,46^. In our study, the cracking-resistant *pg/exp* genotype, had more CSP in the mesocarp portion, and more SSP in the exocarp portion of the fruit. In contrast, there was more WSP in fruit of the WT genotype. This observation suggests that both the exocarp and mesocarp cell walls of *pg/exp* fruit were more intact and thus better able to resist internal stresses that are presumed to promote ripe fruit cracking.

The calcium content of the fruit and their cell walls can affect cell wall strength. Pectins with low levels (<40%) of methoxyl-esterification can form gels; calcium-ion (Ca^2+^) bridging of unesterified GalA residue carboxyl groups on neighboring HG pectins has been proposed to form “egg-box” structures in the primary cell wall matrix^47^. And the strength of the Ca^2+^-promoted-gels increases with increasing Ca^2+^ concentration. In this experiment, the higher Ca^2+^ level in the AIR from WT fruit than in the AIR from the firmer and less-cracked *pg/exp* fruit is somewhat surprising. It is not clear how the AIR's Ca^2+^ content corresponds with the relative distributions of Ca^2+^ in the cell wall/apoplast, the cytoplasm and the vacuolar compartments. However, our immunofluorescence microscopy with JIM5 and JIM7 antibodies revealed that pg/exp fruit had more highly esterified pectins than WT fruit, indicating less capacity for cell wall binding of Ca^2+^. In some cases, there is also less Ca^2+^ in cracking-resistant varieties^48^. We conclude that a ripe fruit with more intact pectins in its primary walls is likely to resist cracking more effectively, as long as a reasonable degree of pectin-pectin bonding (via Ca^2+^ or other cross-linkages) is retained. The correlation analysis demonstrated that crack rate was associated most significantly with the protopectin and cellulose rather than Ca^2+^, which confirms this view.

In our study, we used whole-plant sprays of ABA to increase the tendency of tomato fruit to crack. ABA application can decrease stomatal conductance and leaf transpiration, and increases plant water potential^49^, which results in significantly increased xylemic flow into tomato fruit^23^. This xylemic flow also carries more Ca^2+^ into the fruit as has been reported previously and is evident in the higher Ca^2+^ levels in both whole fruit and cell walls of ABA treated fruit. The higher incidence of cracking in ABA treated RR tomato fruit was likely due to accumulation of water in the fruit when leaf transpiration was reduced by ABA, likely resulting in increases in turgor pressure in the fruit. However, the fruit genotypes showed significant differences in their tendency to crack when cracking was promoted by ABA application. The increase in cracking in response to ABA treatment was not observed in RR *pg/exp* fruit but was observed in WT and *glk2* fruit. And there was no difference in cracking among the three genotypes when plants were treated with water.

ABA treatment also had an influence on tomato cell wall composition, resulting in lower amounts of WSP and SSP in mesocarp and blossom end tissues of all three genotypes. This does not appear to be related to influences on fruit ripening as no visible differences in ripening were observed and ABA is generally reported to enhance ripening, not slow ripening. The higher proportion of chelator soluble cell wall material (CDTA soluble, CSP) may be a response to the higher Ca^2+^ levels in the fruit due to the higher xylemic flow, but it is unclear what role if any this played in the increased cracking of ABA treated fruit.

Cell walls from *pg/exp* fruit also appeared thicker and denser than cell walls from WT fruit under electron microscopy, perhaps because of reduced disassembly of the cell wall polysaccharide polymer network. This difference could be another reason for resistance to cracking in this genotype. The thicker and denser cell walls from *pg/exp* fruit are reflected in the higher levels of CSP, SSP and cellulose in cell wall extracts prepared from the mesocarp of *pg/exp* fruit. Cantu^46^ previously demonstrated that the reduction of both PG and EXP activities resulted in isolated and in situ cell walls that swelled much less than walls from WT fruit that soften significantly at the fully ripe stage; which supports the conclusion that *pg/exp* fruit has a more intact cell wall than WT fruit. In our experiments, *pg/exp* had a thicker and denser cell wall that may resist swelling.

The cuticular wax layer was thicker in *pg/exp* fruit, which could also contribute to resistance to cracking. While waxes are part of the overall extracellular matrix, they are not targets of either PG or EXP, which suggests that suppression of the ripening-associated *SlPG* and *SlEXP1* genes may also have impacts on other structures at the fruit surface. It is interesting to note that in addition to changing cell wall network integrity, tomato fruit cuticle chemistry, and structure have been identified as fruit factors that influence ripening-associated fruit softening^50^.

The correlation analysis also showed that cracking rate was significantly associated with cell wall composition (protopectin and cellulose) and cell wall-thickness, as well as with wax-thickness. Since increased water uptake by the fruit due to exposure of the plants to ABA promotes cracking, the physical constraints of the more intact cell walls in the *pg/exp* fruit seem to be the major factor providing resistance to cracking at the later stage of ripening (RR).

## Conclusion

**Fig. 9:**
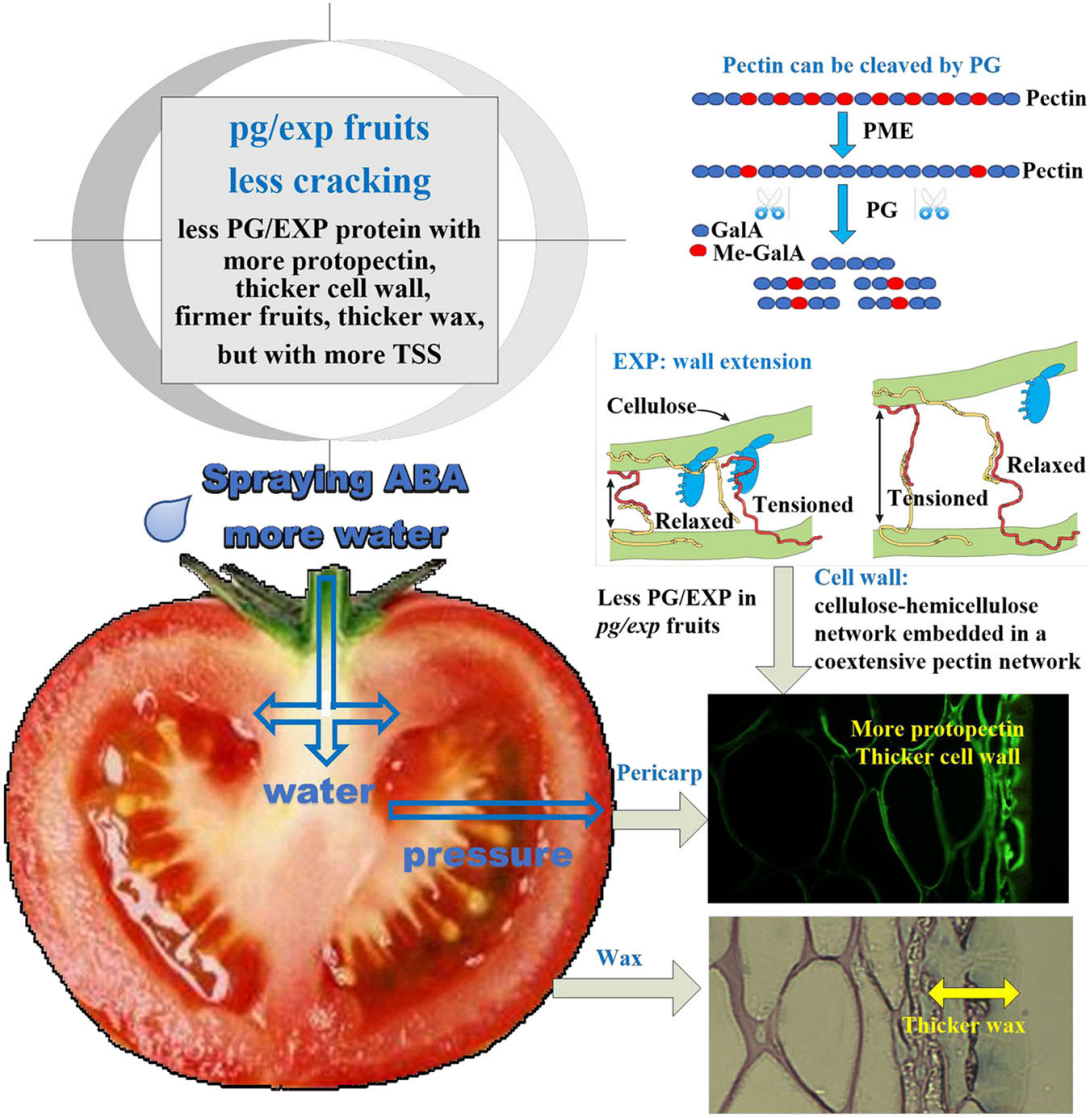
Mechanism of ABA treated *pg/exp* fruit showing less cracking. *pg/exp* indicates transgenic fruit with suppressed *SlPG* and *SlEXP1* expression. ABA treatment of tomato plants was used to increase water flow into fruit. However *pg/exp* fruit showed less cracking than wild type due to less polygalacturonase (SlPG) and expansin (SlEXP1) proteins in fruits. PG and EXP can cooperatively disassemble tomato fruit cell walls. With decreased PG and EXP proteins, *pg/exp* fruit are more firm, have more protopectin, thicker cell walls, and a thicker wax layer, but have more TSS and have less cracking in red ripe fruits

Ripening-related disassembly of the fruit cell wall, but not elimination of SlGLK2, influenced tomato fruit cracking. The simultaneous suppression of SlPG and SlEXP1 in ripening fruits reduced cell wall disassembly; *pg/exp* fruit were more firm, had more protopectin, thicker cell walls and wax, but with more TSS (Fig. 9). We conclude that a ripe fruit with more intact pectins in its primary walls is likely to resist cracking more effectively, as long as a reasonable degree of pectin-pectin bonding (via Ca^2+^ or other cross-linkages) is retained. The correlation analysis demonstrated that crack rate was associated most significantly with protopectin and cellulose content, rather than Ca^2+^, which confirms this view. The complexity of the fruit cell wall and its disassembly provides multiple opportunities to improve ripe fruit characteristics, including limiting losses due to cracking during ripening, handling, and distribution.
